# Cloning, Expression, and Characterization of GDSL-Type Lipolytic Enzyme Genes from *Epidermidibacterium keratini* EPI-7 Isolated from Human Skin

**DOI:** 10.4014/jmb.2504.04022

**Published:** 2025-08-06

**Authors:** Seok-Yun Jeong, Seok Kyun Yun, Suhyeon Cho, Seyeol Baek, Hee-Jae Shin, Seokmuk Park, Sugyeong Jeong, Gayoung Kim, Seunghyun Kang, Seunghee Bae

**Affiliations:** 1 Department of Cosmetics Engineering, Konkuk University, Seoul 05029, Republic of Korea; 2 Department of Biological Engineering, Konkuk University, Seoul 05029, Republic of Korea; 3 COSMAX BTI R&I Center, Seongnam-si 13486, Republic of Korea

**Keywords:** GDSL-type esterase/lipase, heterologous expression, gene cloning, enzyme characterization, *Epidermidibacterium keratini*, skin microbiome

## Abstract

This study investigated seven putative lipolytic enzymes (EstEk01-07) from the skin microbiome bacterium *Epidermidibacterium keratini* EPI-7, focusing on their properties relevant to industrial applications. Sequence analysis revealed conserved GDSL motifs and four conserved blocks, characteristic of the GDSL/SGNH superfamily, with predicted α/β/α folds consistent with these enzymes. Significant variations in the number of α-helices and β-sheets among the EstEk enzymes suggested diverse substrate specificities and catalytic efficiencies. The enzymes exhibited a strong preference for short-chain fatty acids (C2-C4), classifying them as carboxylesterases, a novel finding within the skin microbiome. Optimal enzyme activity was observed at alkaline pH (8.0-9.0) and thermophilic condition (50-60°C), with substantial thermostability retained after heating at 50°C for three hours. Metal ion analysis revealed a significant stimulatory effect of Ca^2+^ and Fe^3+^, while other transition metals were inhibitory. The enzymes were stable in a range of non-ionic detergents, but sensitive to SDS. Moreover, they exhibited notable tolerance to various organic solvents, particularly methanol and isopropanol, suggesting potential applications in cosmetics and pharmaceutical industries. This study identifies a novel library of thermostable, alkaline carboxylesterases from the skin microbiome, highlighting their potential for industrial biocatalysis and further investigation into their role in skin lipid metabolism.

## Introduction

Esterase (E.C.3.1.1.1) and Lipase (E.C.3.1.1.3) are glycerol ester hydrolase that catalyze the hydrolysis of triglycerides into diglycerides, monoglycerides, glycerol, and free fatty acids. Although they resemble each other in catalytic activity, they differ in their preferred substrates. Esterases (E.C.3.1.1.1) catalyze the hydrolysis of ester bonds of water-soluble substrates, with a preference for short-chain carboxylic acid esters, whereas lipases (E.C.3.1.1.3) catalyze the hydrolysis of ester bonds at the interface between hydrophobic phase and the aqueous phase, favoring long-chain carboxylic acid esters [1]. These enzymes are found in various organisms, including animals, plants, and microorganisms. Among them, microbial enzymes are particularly valuable for industrial applications due to their simplicity of genetic manipulation, diverse catalytic activities, rapid growth on economical media, and consistent high yield production [2, 3].

Bacterial lipolytic enzymes are classified into eight different families based on the similarity of their amino acid sequences [4]. Most lipolytic enzymes contain the pentapeptide Gly-Xaa-Ser-Xaa-Gly (GxSxG) motif with the catalytic serine residue located near the center of the conserved sequence. However, not all lipolytic enzymes exhibit the conventional GxSxG motif. The GDSL-type lipolytic enzymes possess the Gly-Asp-Ser-Leu (GDSL) motif containing the catalytic serine site situated near the N-terminus, which differs from the classical GxSxG motif in the center of the sequence. Additionally, some members of GDSL family were classified into the SGNH hydrolase family, characterized by four conserved residues Ser, Gly, Asn, and His located in four conserved blocks I, II, III, and V, respectively. Proteins in the SGNH hydrolase family have broad substrate specificities due to the flexibility of their active sites [5]. To date, these enzymes have been identified in various plants and bacteria, and their function has been characterized [6, 7]. However, the reports on characterization and industrial application of SGNH/GDSL hydrolase family proteins derived from skin microbiomes remain limited.

*Epidermidibacterium keratini* EPI-7, which belongs to the *Sporichthyaceae* family, is a skin flora isolated from human epidermal keratinocytes with aerobic, gram-positive, non-motile, and non-spore forming properties. The polar lipids of strain EPI-7 were found phosphatidyl-ethanolamine (PE), phosphatidylinositol (PI), phosphatidyl-glycerol (PG), phosphatidylcholine (PC), and three unidentified phospholipids (UPLs) [8]. Previous studies have shown that fermentation filtrate of EPI-7 with orotic acid showed the effect of enhancing skin barrier function, improving skin elasticity, and dermal density by restoring skin symbiotic microbial diversity [9]. In addition, the culture medium of EPI-7 and 1,1'-biuracil derived from EPI-7 showed protective effects against UV irradiation in HDF (Human Dermal Fibroblast) [10]. It has been demonstrated that the ferments and derived substances from EPI-7 have beneficial effects on the skin, which has prompted numerous studies to explore the potential applications of EPI-7.

Until now, there are few reports about the gene cloning and characterization of lipolytic enzymes from *Epidermidibacterium* strains isolated from human epidermal keratinocytes. In this study, genes encoding GDSL-type lipolytic enzymes from *E. keratini* EPI-7 were cloned and heterologously expressed in *E. coli* BL21. Subsequently, the recombinant enzymes were purified, and the biochemical characterization was studied for its industrial application.

## Materials and Methods

### Materials

The pTrcHis vectors were purchased from Thermo Fisher Scientific (USA). The pKRX-T vector was kindly provided by Prof. Jae Ho Lee (Konkuk University, Republic of Korea) [11, 12]. The APrep Total DNA BYC mini kit for genomic DNA isolation of *E. keratini* EPI-7 was purchased from AP BIOTECH (Republic of Korea). The Pierce BCA Protein Assay Kit for protein quantification was purchased from Thermo Fisher Scientific. Ni-NTA Agarose was purchased from QIAGEN (Germany). The Ex Taq DNA polymerase and dNTP for polymerase chain reaction (PCR) were purchased from TaKaRa (Japan) and BIONEER (Republic of Korea). All restriction enzymes, T4 DNA ligase and reaction buffers used in this study were purchased from NEB (USA). The His-Tag Antibody (H-3), used to detect and analyze protein expression, was purchased from Santa Cruz Biotechnology (USA). All *p*-nitrophenyl synthetic substrates and other reagents used in this study were purchased from Sigma-Aldrich (USA).

### Microorganism and Culture Conditions

*E. keratini* EPI-7 was kindly provided by the COSMAX BTI (Republic of Korea). *E. keratini* EPI-7 was cultured in R2A medium (dextrose 0.5 g, yeast extract 0.5 g, soluble starch 0.5 g, casamino acids 0.5 g, proteose peptone 0.5 g, sodium pyruvate 0.3 g, magnesium sulfate, 0.05 g, dipotassium phosphate, 0.3, per liter, pH 7.2) at 28°C with aeration [8]. *Escherichia coli* TOP10 and BL21 Competent cells were purchased from Thermo Fisher Scientific and used as cloning hosts and expression hosts, respectively. *E. coli* strains were cultured in Luria-Bertani medium (LB, NaCl 5 g, tryptone 10 g, yeast extract 5 g, per liter, pH 7.0) at 37°C with aeration.

### Amino Acid Sequence Analysis and Protein Modeling of GDSL-Type Lipolytic Enzyme Genes

Amino acid sequence analysis and multiple sequence alignment were conducted by Geneious.24.0.3. The secondary structure of each protein was predicted using the Predict Secondary Structure application from Geneious.24.0.3 [13]. To identify potentially secreted enzymes among the seven candidates, signal peptide prediction was performed using SignalP 6.0 (https://services.healthtech.dtu.dk/services/SignalP-6.0/) [14]. This tool distinguishes between Sec/SPI-, Tat/SPI-, and other signal peptide types, allowing for classification of putative extracellular enzymes based on their N-terminal signal sequences. Tertiary structure models were generated using the Phyre2 protein homology modeling server [15], and the resulting structures were visualized and edited using PyMOL 3.0 (https://www.pymol.org/) [16].

### Construction of the GDSL-Type Lipolytic Enzyme Genes Expression Vector

The cells of *E. keratini* EPI-7 were harvested by centrifugation at (4,000 ×*g*, 20 min, 4°C). To obtain PCR templates, the genomic DNA of *E. keratini* EPI-7 was isolated by performing APrep Gram Positive Bacteria DNA isolation protocol. The amplification was performed by PCR with EX Taq DNA polymerase [17-19]. The conditions of PCR were as follows: one cycle of denaturation at 94°C for 5 min, 30 cycles of denaturation at 94°C for 30 sec, annealing temperature (Table 1) for 30 sec, extension at 72°C for 40 sec (EstEk04 was performed for 1 min) and final extension step at 72°C for 5 min. The amplification fragments were recovered by gel extraction and then ligated into the Xcm1 site of the pKRX-T vectors. Ligation was performed using a 1:5 molar ratio of backbone to insert, and the reaction was incubated overnight at 16°C with T4 DNA ligase and 10× ligase buffer (NEB). The recombinant plasmids were transformed into *E. coli* TOP10 competent cells using heat shock transformation protocol. The transformants were plated onto LB plate containing ampicillin (100 μg/ml) and incubated at 37°C overnight. After the preparation of recombinant plasmids, the constructed plasmids were cleaved with restriction enzymes (listed in Table 1) and the digested fragments were ligated into the pTrcHis vector previously digested with the same restriction enzymes. These recombinant expression plasmids were transformed into *E. coli* BL21 for heterogenous expression of the interest genes.

### Purification of Recombinant GDSL-Type Lipolytic Enzyme

The recombinant *E. coli* BL21 cells were cultured in LB broth with ampicillin (100 μg/ml), overnight at 37°C. The cultured cells (2 ml) were transferred to 200 ml LB broth with ampicillin and cultured aerobically at 37°C, 150 rpm. When the OD_600_ of the bacterial cultures reached 0.5-0.6, isopropyl β-D-1-thiogalactopyranoside (IPTG) was added to a final concentration of 0.1 mM to induce protein expression. After induction with 0.1 mM IPTG for 20 h at 20°C, the cells were harvested by centrifugation (3,500 ×*g*, 20 min, 4°C) and washed twice with cold PBS (Phosphate Buffered Saline, pH 7.4). The cells were resuspended in a lysis buffer (50 mM NaH_2_PO_4_, 300 mM NaCl, 10 mM imidazole, pH 7.4) with Lysozyme (1 mg/ml), and incubated on ice for 30 min. For complete cell lysis, the cells were sonicated on ice using a Vibra-Cell VC750 ultrasonic processor (Sonics & Materials, USA) with a net sonication time of 2 min 30 sec at 30% duty cycle (2 sec on-8 sec off), followed by centrifugation at 15,000 ×*g* for 30 min at 4°C to remove insoluble proteins. The supernatants containing the recombinant protein were loaded onto a pre-equilibrated Ni-NTA agarose affinity column. After washing the column with a wash buffer (50 mM NaH_2_PO_4_, 300 mM NaCl, 50 mM imidazole, pH 7.4), the 6x-Histidine-tagged recombinant proteins were eluted with elution buffer (50 mM NaH_2_PO_4_, 300 mM NaCl, 250 mM imidazole, pH 7.4). The elution buffer was subsequently exchanged with 10 mM Tris-HCl buffer (pH 7.4) with 150 mM NaCl by Ultrafiltration (Vivaspin 20 centrifugal concentrator, MWCO 10 kDa; SIGMA, USA). The purity and concentration of the protein samples were analyzed by SDS-PAGE and Bicinchoninic acid (BCA) assay kit [20], respectively. Detection of His-tagged EstEk01-07 proteins was carried out by western blot analysis. A monoclonal His-Tag antibody (H-3, 1:1000) and a goat anti-mouse IgG (H+L) secondary antibody (1:1000; Cell Signaling Technology, USA) were used. Membranes were incubated with the primary antibody overnight at 4°C, followed by incubation with the secondary antibody. After extensive washing, protein expression was detected using an enhanced Clarity MAX Western chemiluminescence (ECL) substrate reagent (Bio-Rad, USA) and visualized with the ChemiDoc Touch Imaging System (Bio-Rad).

### Periplasmic fraction of Recombinant GDSL-Type Lipolytic Enzyme

The periplasmic fraction was prepared using the PureFrac method [21]. After induction with 0.1 mM IPTG for 20 h at 20°C, the recombinant cells were harvested by centrifugation (3,500 ×*g*, 20 min, 4°C) and washed with cold PBS. The cell pellet was resuspended in 900 μl of spheroplast buffer (0.1 M Tris, 0.5 M sucrose, 0.5 mM EDTA, pH 8.0), and incubated for 5 min. After centrifugation, the supernatant was carefully removed. The resulting pellet was then resuspended in 400 μl of distilled water containing 1 mM MgCl_2_, incubated on ice for 15 sec, followed by the addition of 20 μl 20 mM MgSO_4_. After centrifugation, the periplasmic fraction was carefully collected and analyzed by SDS-PAGE and western blot.

### Enzyme Activity and Substrate Specificity Assay

Lipolytic enzyme activity was measured spectrophotometrically using *p*-nitrophenyl (*p*-NP) esters with alkyl chain as substrates [22, 23]. The standard assay was carried out at 40°C for 15 min in a 100 μl reaction mixture containing 10 μl of *p*-NP esters dissolved in iso-propanol (10 mM), 90 μl of Tris-HCl buffer (50 mM Tris-HCl, pH 8.0, 0.4% Triton X-100, and 0.1% gum arabic), and 1 μg of purified enzyme. The enzymatic reactions were stopped by a direct chilling method [24]. To correct for non-specific substrate hydrolysis, blank reactions were performed using enzyme-free elution buffer (10 mM Tris-HCl, pH 7.4, containing 150 mM NaCl) under identical conditions. The resulting blank values were subtracted from the test reactions to eliminate background hydrolysis not attributable to enzymatic activity. The liberated *p*-NP was quantified at 410 nm using a microplate spectrophotometer (Synergy HTX, BioTek, USA). Under the above conditions, the molar extinction coefficient of *p*-NP was 1.72 × 10^4^ M^-1^ cm^-1^ at 410 nm. One unit (1 U) of lipolytic enzyme activity was defined as the amount of enzyme required to liberate 1 μmol *p*-NP per minute at 40°C. The substrate specificity of the lipolytic enzyme was determined on *p*-NP esters with different alkyl chain length (C2-C16) under the conditions described above. All measurements were performed in triplicate.

### Effect of pH and Temperature on the Enzyme Activity and Stability

To determine the optimal pH of the enzyme, enzyme activity was assayed at 40°C for 15 min using 1mM *p*-NP C2 (EstEk01-06) or *p*-NP C4 (EstEk07) as substrate in different buffers ranging from pH 5 to 10: 50 mM sodium acetate (pH 5.0-6.0); sodium phosphate (pH 6.0-8.0); Tris-HCl (pH 8.0-9.0); Glycine/NaOH (pH 9.0-10.0). For pH stability, the purified enzymes were pre-incubated at room temperature for 3 h in various pH buffers, and the residual activity was then measured under standard conditions.

To evaluate the optimal temperature of the enzyme, enzyme activity was measured at the temperature ranging from 0°C to 80°C for 15 min in 50 mM sodium phosphate buffer (pH 8.0) using 1 mM *p*-NP C2 (EstEk01-06) or *p*-NP C4 (EstEk07) as substrate. For thermostability, the purified enzymes were pre-incubated at the temperature range from 10°C to 80°C for 3 h, and the residual enzyme activity was then measured under standard conditions. All measurements were performed in triplicate.

### Effects of Metal Ions, Detergents, and Inhibitors on the Enzyme Activity

The effects of metal ions, inhibitors, and detergents on the enzyme activity were carried out by pre-incubating the recombinant enzyme in Tris-HCl buffer (pH 8.0) containing 1 mM and 10 mM metal ions (Co^2+^, Fe^3+^, Ni^2+^, Cu^2+^, K^+^, Na^+^, Al^3+^, Mg^2+^, Zn^2+^, Ca^2+^, and Mn^2+^), 1 mM and 10 mM inhibitors (EDTA, and PMSF), and 1% detergents (SDS, Triton X-100, Tween-20, and Tween-80) at room temperature for 1 h. The reaction was initiated by adding the substrate, and the residual enzyme activity was measured under standard conditions. Blank reactions were conducted under identical conditions without enzyme. The enzyme activity of a control, incubated under the same conditions without metal ions, detergents, and inhibitors, was taken as 100%. All measurements were performed in triplicate.

### Effects of Organic Solvents on the Enzyme Activity

The effect of organic solvents on the enzyme activity was evaluated by measuring the residual enzyme activity after incubating the enzyme in Tris-HCl buffer (pH 8.0) containing 15% (v/v) of various solvents (methanol, ethanol, iso-propanol, acetone, acetonitrile, DMSO) at room temperature for 1 h. The residual activity was then measured under standard conditions. Blank reactions were carried out under identical conditions without enzyme. Enzyme activity in the control, incubated under the same conditions without organic solvents, was set to 100%. All measurements were performed in triplicate.

### Statistical Analysis

All data in this study are presented as means ± standard deviations (SDs) from three independent experiments conducted for each group. Statistical analyses were performed using one-way ANOVA, followed by Tukey’s post hoc test, as indicated in the figure legends. Statistical significance was indicated as ^*^ or ^#^ for *p* < 0.001.

## Results and Discussion

This study aimed to clone and characterize GDSL-type lipolytic enzyme genes from *E. keratini* EPI-7 and to explore their potential applications. Seven novel genes, designated EstEk01 to EstEk07, were identified from the genome, amplified, and heterologously expressed in *E. coli* BL21. As *E. keratini* EPI-7 is a skin commensal bacterium associated with barrier function, its lypotic enzymes were investigated to elucidate their role in lipid metabolism. The biochemical and structural properties of the seven enzymes were examined, including their sequences, predicted structures, enzymatic activities, and stability profiles.

### Lipolytic Activity of Strain *Epidermidibacterium keratini* EPI-7

**Fig. 1:**
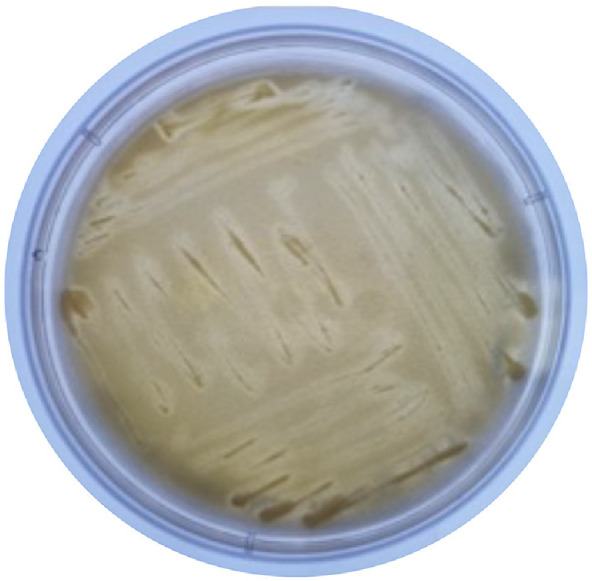
Lipolytic activity of *E. keratini* EPI-7. Clear zones were formed around *E. keratini* EPI-7 colonies on LB agar containing 1% tributyrin.

The human skin surface hosts a complex microbial community that interacts with various substrates, including fatty acid, impacting skin homeostasis and disease susceptibility [25]. Esterases are vital in fatty acid metabolism on the skin surface, influencing lipid composition and contributing to epidermal barrier function [26]. Epidermal lipids, especially in the outermost layer, affect keratinocyte proliferation and differentiation, thereby impacting skin barrier function and contributing to various skin conditions [27]. However, the relationship between the resident skin microbiota and surface lipid metabolism remains largely unexplored. The strain of *E. keratini* EPI-7 (GenBank ID: NZ_CP047156), an actinobacterial strain previously reported, is gram-positive, non-motile, and grows within a temperature range of 15–35°C, with 25°C as the optimum growth temperature [8]. EPI-7 was isolated from human epidermal keratinocytes and was kindly provided by the COSMAX BTI (Republic of Korea). We assessed its lipolytic activity by observing the formation of clear zones around colonies grown on tributyrin (TBN) LB plates (Fig. 1). EPI-7 exhibited a hydrolysis halo in agar plate medium supplemented with tributyrin.

### Sequence Analysis of Putative Lipolytic Enzyme Genes

GDSL/SGNH esterases, known for their versatile catalytic capabilities, can hydrolyze ester bonds and, under specific conditions, performing synthetic or stereoselective reactions [28]. They are also recognized for their structural flexibility and stability under various environmental conditions [29]. Based on the genomic sequence of *E. keratini* EPI-7, seven putative GDSL/SGNH-type lipolytic enzyme genes were identified through domain-based sequence analysis, independent of halo-based activity screening. These genes were annotated as "GDSL-type esterase/lipase family protein" (EstEk01; WP_159546105, 774 bp), and "SGNH/GDSL hydrolase family protein" for EstEk02 (762 bp), EstEk04 (1,488 bp), EstEk05 (921 bp), and EstEk07 (702 bp). Additionally, EstEk03 (1,113 bp; WP_225983862) and EstEk06 (792 bp; WP_159546432) were found to contain overlapping ORFs.

**Fig. 2:**
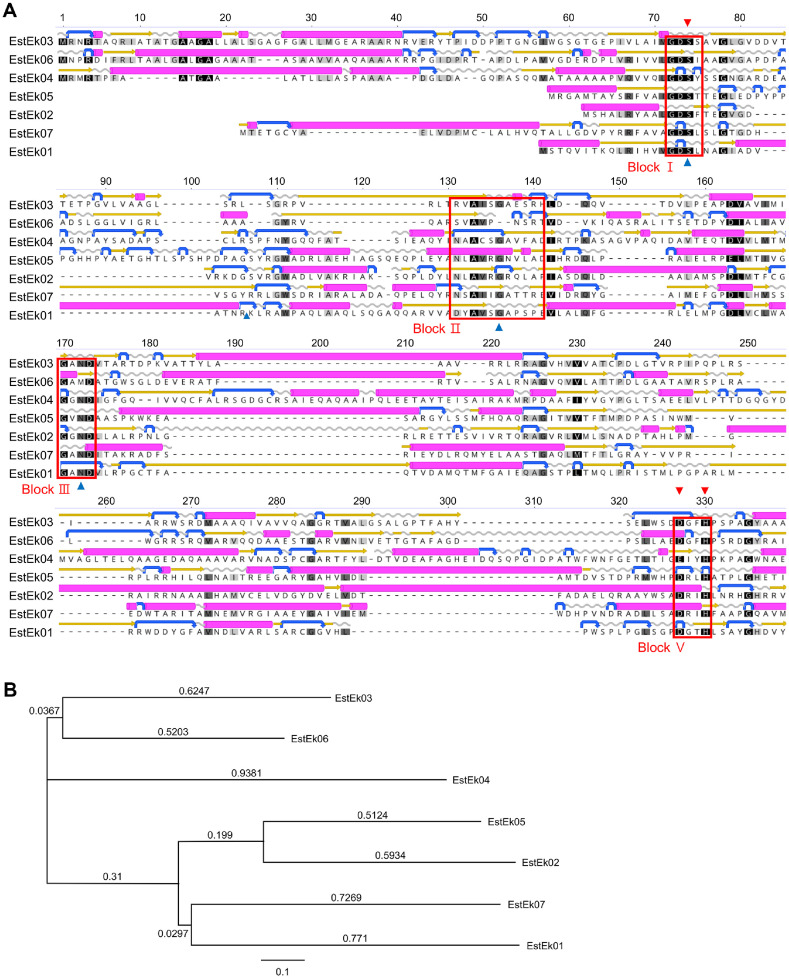
Multiple amino acid sequence alignment (A) and phylogenetic analysis (B) of EstEk01-07 from *E. keratini* EPI-7. (**A**) The purple bars represent α-helix structure, the yellow arrows represent β-strand, and the blue arrows represent Turn. Four conserved blocks of I, II, III, and V are boxed. The black and gray blocks show the identical and similar sequences, respectively. Closed triangles and closed inverted triangles indicate amino acid residues belonging to oxyanion hole, and the catalytic triad, respectively. (**B**) Phylogenetic tree of EstEk01-07 from *E. keratini* generated using Geneious Prime 24.0.3. Sequence with the following accession numbers were obtained from NCBI/GenBank: *E. keratini* EPI-7 (CP047156). EstEk01: GDSL-type esterase/lipase family protein of *E. keratini* EPI-7 (Accession No. WP_159546105), EstEk02-07: SGNH/GDSL hydrolase family protein of *E. keratini* EPI-7 (Accession No. WP_225983817, 225983862, 159544411, 159544506, 159546432, and 159544982, respectively).

As shown by Fig. 2A and 2B, amino acid sequences of the seven enzymes were aligned using Geneious Prime 24.0.3. All sequences featured a GDSL-like motif (Gly-Asp-Ser-Leu-X) near the N-terminus (block I), along with four conserved sequence blocks (I, II, III, and V) containing the conserved residues Ser, Gly, Asn, and His, characteristic of the GDSL/SGNH superfamily. These structural variations highlight the diversity and reactivity of SGNH/GDSL enzymes, which contain a catalytic triad of Ser, Asp, and His, as well as essential residues such as Gly in Block II-crucial for forming oxyanion holes-and Asn in Block III, which is important for substrate stabilization [30]. Notably, the absence of the GxSxG motif (nucleophilic elbow) distinguishes these enzymes from classical α/β-hydrolases.

To assess the secretion potential of these enzymes, signal peptide prediction was performed using SignalP 6.0 (Table S1). The analysis indicated that EstEk03, EstEk04, and EstEk06 contain N-terminal signal peptides, suggesting their secretion through the Sec or Tat pathways. In contrast, the remaining four enzymes (EstEk01, EstEk02, EstEk05, and EstEk07) lack detectable signal peptides, implying that they are likely retained in the cytoplasm.

### Three- Dimensional(3D) Structure and Modeling

**Fig. 3:**
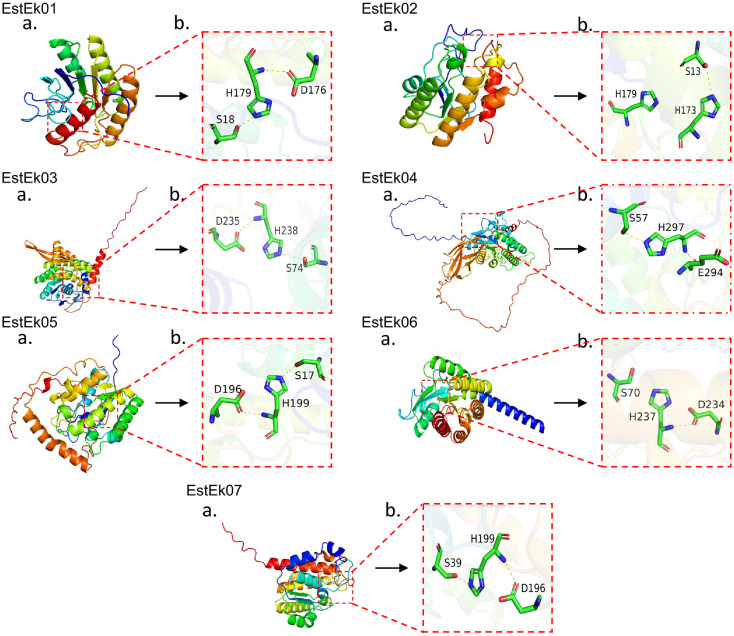
3D Structure Modeling of EstEk01-07. (**A**) The α-helix, β-sheet, and random coil are shown in cartoon. (**B**) The catalytic triad are zoomed in.

The 3D structural model of EstEk01 was generated using Phyre2, based on its most similar structure, acyl-CoA thioesterase I (PDB ID: 5TIE). Similarly, the structures of EstEk02 (PDB: 8H09), EstEk03 (PDB: 4XVH), EstEk04 (PDB: 4HYQ), EstEk05 (PDB: 1YZF), EstEk06 (PDB: 3KVN), and EstEk07 (PDB: 1YZF) were constructed using their respective PDB entries as templates, all of which showed over 99% confidence (Fig. S1). These results suggest that enzymes may function as hydrolases capable of hydrolyzing ester bonds and structurally related linkages. Further comparative analysis revealed that the predicted 3D structures of these enzymes, compared to previously analyzed microbial hydrolases, display an α/β/α fold, confirming their membership in this enzymatic family. This fold is characterized by α-helices flanking a central parallel β-sheet, contributing to the enzyme’s stability, substrate binding, and catalytic activity [31]. As shown in Fig. 3A and 3B, EstEk01 contains five α-helices and five β-sheets; EstEk02 contains six α-helices and five β-sheets; EstEk03 contains eleven α-helices and eight β-sheets; EstEk04 contains thirteen α-helices and eight β-sheets; EstEk05 contains seven α-helices and four β-sheets; EstEk06 contains eight α-helices and four β-sheets; and EstEk07 contains seven α-helices and four β-sheets. These variations in the number of α-helices and β-sheets among the EstEk enzymes may contribute to differences in substrate specificity and catalytic efficiency.

### Expression, Purification, and Periplasmic Fraction of the GDSL-Type Lipolytic Enzymes

**Fig. 4:**
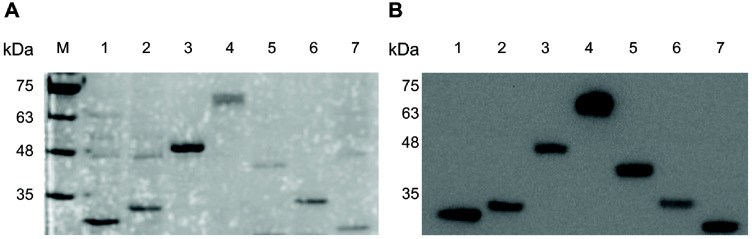
SDS-PAGE and Western blot analysis of recombinant EstEk01-07 proteins from *E. keratini* EPI-7. (**A**) SDS-PAGE analysis of the purified EstEk01-07. (**B**) Western blotting was performed using an anti-His antibody to detect EstEk01-07. Lane M, molecular size marker; lane 1, purified EstEk01; lane 2, purified EstEk02; lane 3, purified EstEk03; lane 4, purified EstEk04; lane 5, purified EstEk05; lane 6, purified EstEk06; lane 7, purified EstEk07.

Expression of the lipolytic enzyme genes was driven by the Trc promoter and induced by the addition of 0.1 mM IPTG at 20°C for approximately 20 h. The crude enzyme extracted from recombinant *E. coli* BL21 cells was purified by Ni-NTA agarose affinity column. Expression and purification were confirmed by Coomassie Brilliant Blue staining. The total cell lysate, the soluble fraction, and the elution fraction were collected and analyzed by 10% SDS-PAGE (Fig. S2). To verify the expression of the target proteins, western blotting was performed using an anti-His antibody. As shown in Fig. 4, the deduced molecular mass of EstEk01, based on the open reading frame (ORF), was calculated to be 27.6 kDa, corresponding to 31.7 kDa with the addition of the N-terminal 6x-His tag. Similarly, the predicted molecular weights for EstEk02 to EstEk07 were 27.5, 38.8, 50.2, 33.6, 26.9, and 25.6 kDa, with predicted values of 32.7, 43.6, 54.6, 38.7, 31.5, and 30.2 kDa, respectively, including the 6x-His tag. All recombinant enzymes were detected near the predicted molecular weights, except for EstEk04, which was observed at approximately 66 kDa.

Based on the SignalP 6.0 predictions, periplasmic fractionation was performed for recombinant *E. coli* BL21 expressing EstEk03, EstEk04, and EstEk06 using the PureFrac method, followed by SDS-PAGE and western blot analysis. As shown in Fig. S3, the enzymes were detected in the periplasmic fraction. These findings support the hypothesis that only a subset of GDSL/SGNH-type enzymes from *E. keratini* EPI-7 are secreted and may contribute to the extracellular lipolytic activity observed in the native strain. Notably, EstEk04 contains a predicted LPXTG cell wall anchor motif [32], which may facilitate its localization on the cell surface and contribute to halo formation on tributyrin agar plates. However, due to fundamental differences in membrane architecture between Gram-positive and Gram-negative bacteria, heterologous expression in *E. coli* may not accurately replicate the native secretion behavior, potentially limiting extracellular enzyme activity in the recombinant host [33].

### Substrate Specificity of the GDSL-Type Lipolytic Enzymes

**Fig. 5:**
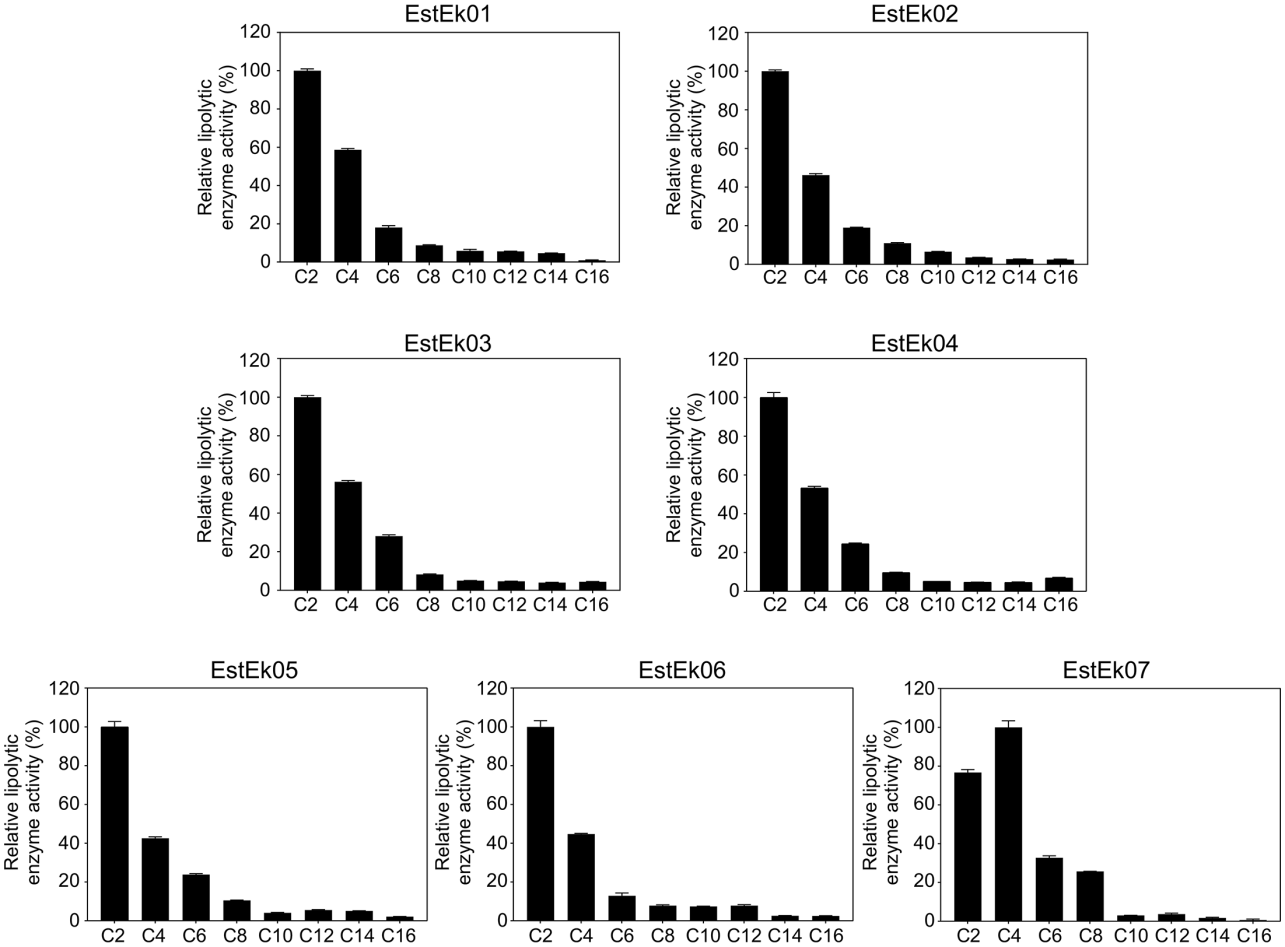
Substrate specificity of recombinant EstEk01-07. The lipase activity of the purified recombinant enzymes toward various chain lengths of *p*-NP esters was assayed at 40°C with final substrate concentrations of 10 mM in 50 mM Tris- HCl buffer, pH 8.0. The highest level of activity with the substrate was taken as 100%. The error bars represent the means ± SD (*n* = 3). C2, *p*NP-C2; C4, *p*NP-C4; C6, *p*NP-C6; C8, *p*NP-C8; C10, *p*NP-C10; C12, *p*NP-C12; C14, *p*NP-C14; C16, *p*NP-C16.

A colorimetric lipolytic enzyme activity assay using *p*-NP esters of different chain length (C2-C16) was performed to determine the substrate specificity of seven putative lipolytic enzymes (Fig. 5). The recombinant GDSL-type lipolytic enzymes from *E. keratini* EPI-7 exhibited a strong preference for short-chain fatty acids (C2-C4). However, no significant lipolytic enzyme activity was observed with medium-chain fatty acids (C6-C12), and no activity was detected with long-chain fatty acids (C14-C16). The specific activities of purified recombinant EstEk01–EstEk07 were measured using *p*-NP C2 (and *p*-NP C4 for EstEk07) as the substrate at pH 8.0 and 40°C, ranging from 0.924 to 2.85 U/mg. These variations may reflect differences in protein expression levels, folding efficiency, or proteolytic degradation.

Based on the substrate preference profile, the GDSL-type lipolytic enzymes from *E. keratini* EPI-7 could be classified as carboxylesterase, consistent with their established role in hydrolyzing short chain esters [34]. This finding highlights a distinct substrate preference within the GDSL/SGNH lipase family, potentially reflecting adaptation to the specific lipid environment of the skin’s surface. This study presents the first comprehensive characterization of a library of GDSL-type lipolytic enzymes from the bacteria-based skin microbiome. The lack of previously reported studies on similar enzymes within this microbial community suggests a unique or novel functional adaptation of these enzymes within the human skin ecosystem. While studies on bacterial lipases in the skin microbiome are limited, various lipases have been reported in the fungal microbiota found on the human scalp, specifically *Malassezia* species. These lipases, including *MrLIP* and *MgLIP1*, have been implicated in Malassezia-associated conditions like hair loss, seborrheic dermatitis related to lipid abnormalities, and disruption of skin barrier integrity [35]. Furthermore, *Staphylococcus epidermidis*, a common skin commensal, produces sphingomyelinase, a lipid hydrolase that affects the metabolism of long-chain fatty acids, including sphingolipids, thereby influencing skin barrier integrity [36]. The role of short-chain fatty acids (SCFAs) in modulating skin barrier function and inflammation has also been reported [37]. These findings collectively suggest a strong association between lipolytic enzymes secreted by skin microbiome members and skin health, indicating a potential role for the carboxylesterase-producing *E. keratini* EPI-7 skin-related processes. The specific interplay between these enzymes and the major fatty acids presents on human skin, as well as seasonal variations in specific fatty acid utilization, requires further investigation.

### Effect of pH and Temperature on the Enzyme Activity and Stability

**Fig. 6:**
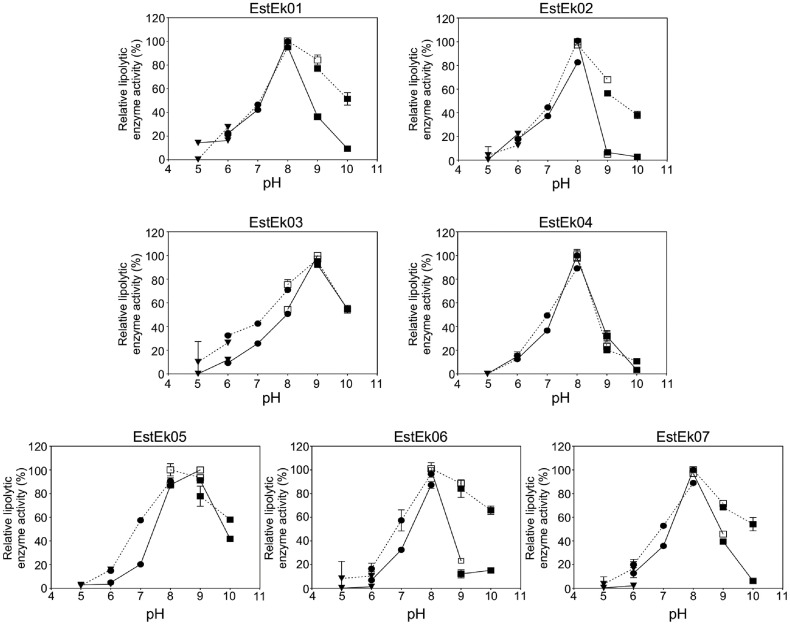
Effect of pH on recombinant EstEk01-07 activity and stability. For the determination of optimal pH, the activity was measured by hydrolyzing *p*-NP-C2 at 40°C for 15 min in different buffers ranging from pH 5 to 10 (solid line). The highest level of activity with the substrate was taken as 100%. The pH stability of enzymes was assayed by pre-incubating the enzymes at different buffers of pH 5.0-10.0 for 3 h at room temperature (dashed line). Sodium acetate buffer (▼, pH 5.0-6.0), sodium phosphate buffer (●, pH 6.0-8.0), Tris-HCl buffer (□, pH 8.0-9.0), and glycine/NaOH buffer (■, pH 9.0-10.0). The activity at time 0 for each pH condition was considered 100%. The error bars represent the means ± SD (*n* = 3).

The optimal pH for the lipolytic enzyme activity of EstEk01-06 was determined using *p*-NP C2 as a substrate, in 50 mM buffer ranging from pH 5.0 to pH 10.0, at 40°C. For EstEk07, the experiment was conducted using *p*-NP C4 as a substrate under the identical conditions. As shown in Fig. 6, EstEk (01-02, 04, and 06-07) exhibited optimal activity at pH 8.0, with a significant decrease in lipolytic activity observed below pH 7.0 and above pH 9.0. EstEk03 and EstEk05 showed optimal activity at pH 9.0, with a marked reduction in activity below pH 7.0. Notably, EstEk03 retained over 50% of its maximum activity at pH 8.0 and 10.0. Also, EstEk05 maintained more than 50%of its maximum activity at pH 7.0-10.0. In the pH stability study, EstEk01-07 remained stable at pH 8.0-9.0, remaining over 70% residual activity after 3 h of incubation, except for EstEk04, which retained approximately 20% at pH 9.0. These results indicate that EstEk01-07 are alkaline enzymes [38].

**Fig. 7:**
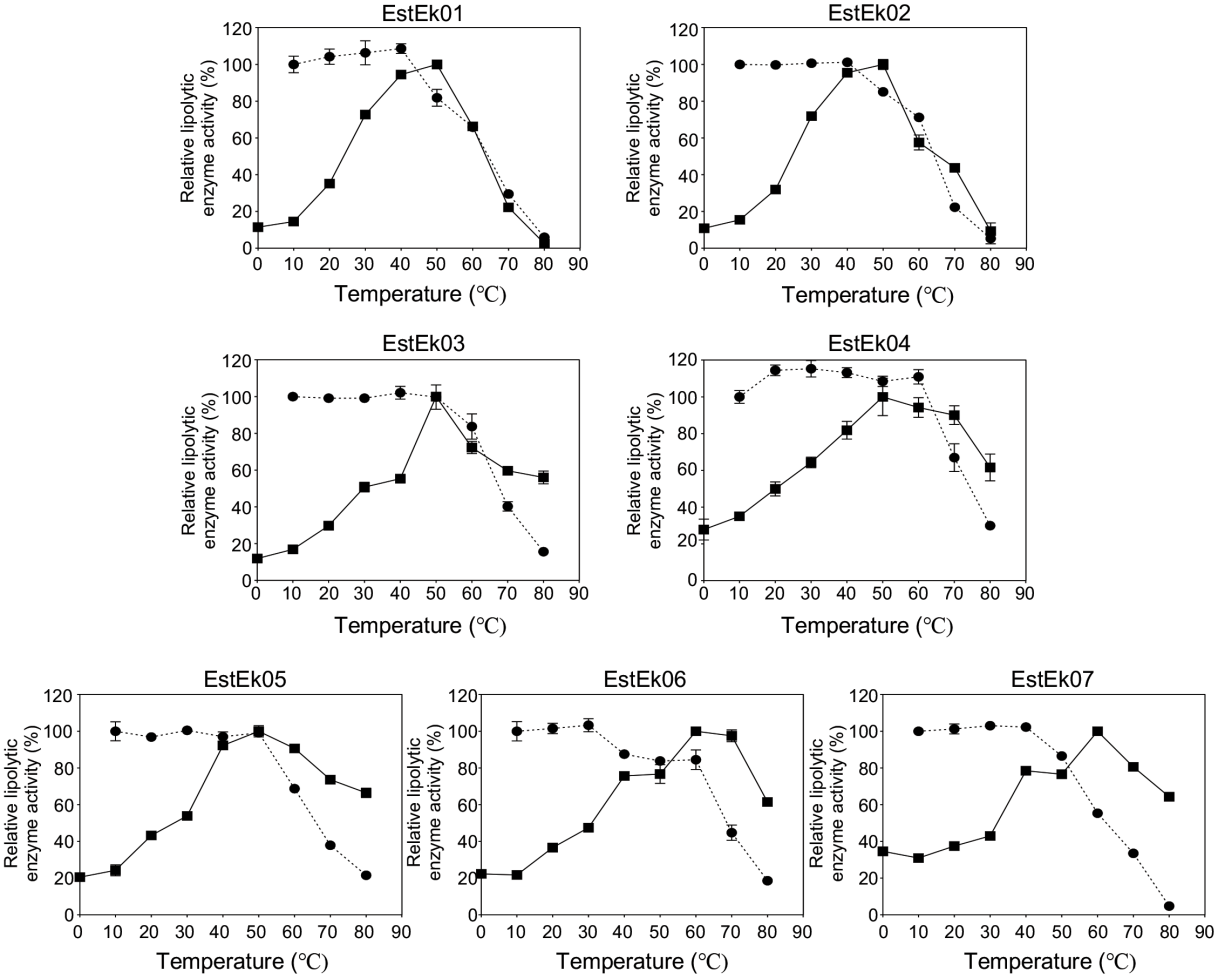
Effect of temperature on recombinant EstEk01-07 activity and thermostability. For the determination of optimal temperature, the activity was measured in Tris-HCl buffer (pH 8.0) at different temperatures ranging from 0°C to 80°C (solid line). The highest activity observed was defined as 100%. The thermostability of enzymes was assayed by pre-incubating enzymes at different temperatures (10-80°C) for 3h and measured under standard conditions (dashed line). The relative activity of the enzyme maintained at 10°C was taken as 100%. The error bars represent the means ± SD (*n* = 3).

The optimum temperature for the lipolytic activity of EstEk01-07 was determined using *p*-NP C2 as a substrate over a temperature range of 0-80°C in 50 mM Tris-HCl buffer at pH 8.0. For EstEk07, the experiment was performed under the same conditions using *p*-NP C4 as a substrate. As shown in Fig. 7, EstEk01-05 exhibited maximum activity at 50°C, while EstEk06-07 showed maximum activity at 60°C. The activity of EstEk01-07 decreased sharply at temperature below 30°C. Notably, EstEk03-07 maintained over 50% of their maximum enzyme activity across a broad temperature range (40-80°C), with the exception of EstEk01-02. To evaluate thermostability, purified EstEk01–07 enzymes were incubated at temperatures ranging from 10°C to 80°C for 3 h. All enzymes retained over 80% of their activity after incubation at 50°C for 3 h, but showed gradual inactivation above 60°C. At 70°C for 3 h, EstEk01–07 maintained 20–40% residual activity. This thermostability surpasses that of previously reported GDSL-type esterases, such as the enzyme from *Geobacillus thermoactenulatus* KCTC3921, which loses activity after heating at 70°C for 1 h [7], and alkaline SGNH hydrolase from Bacillus sp. K91, which is inactivated after incubating at 65°C for 1h [39]. In particular, EstEk04 retained approximately 60% of its activity after heating at 70°C for 3h.

These results indicate that EstEk01-07 exhibit high thermostability and alkaline activity, suggesting their potential applicability across various industries [40]. The pH and temperature optima of EstEk01-07 are comparable to those of S2 and S11 lipases from *S. epidermis*, which exhibit optimal activity at pH 8.0-9.0 [41]. However, while S2 and S11 lipases exhibit maximal activity at 32°C and lose activity rapidly after incubation above 55°C for 30 min, the carboxylesterases from *E. keratini* EPI-7 demonstrate notable thermostability, retaining over 80% activity after heating at 50°C for 3 h. This thermostability is particularly notable given their origin in the skin microbiome, which is typically exposed to moderate temperature ranges. Such stability significantly enhances their potential for industrial applications compared to previously characterized GDSL enzymes from other sources, enabling their use under broader processing conditions beyond typical laboratory settings.

### Effect of Metal Ions, Inhibitors, Detergents, and Organic Solvents on the Enzyme Activity

**Fig. 8:**
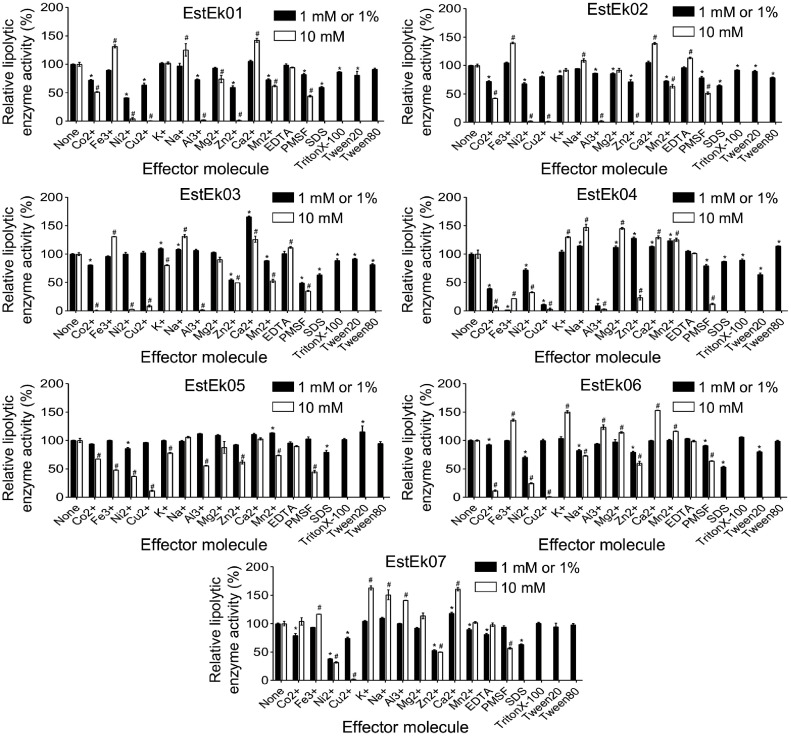
Effect of metal ions, inhibitors, and detergents on recombinant EstEk01-07. The enzymes were preincubated with metal ions, (1 mM, and 10 mM), inhibitors (1 mM, and 10 mM), and detergents (1%) at room temperature for 1 h, respectively. Residual activity was then assayed under standard conditions. The activity of the enzyme without additives was defined as 100%. The error bars represent the means ± SD (*n* = 3). Statistical significance was determined by one-way ANOVA followed by Tukey’s post hoc test. ^*^ or ^#^ indicates *p* < 0.001 compared with the control group.

To investigate the effects of metal ions, inhibitors, and detergents on the activity of EstEk01-07, purified enzymes were pre-incubated with 1 mM or 10 mM metal ions, and inhibitors, and with 1% detergents at room temperature for 1 h. As shown in Fig. 8, Ca^2+^ stimulated or maintained the activity of EstEk01–07 at both concentrations, with activity reaching 100–165% at 10 mM Ca^2+^. Similarly, Fe^3+^ maintained or enhanced activity (90–140%), except for EstEk04 and EstEk05, which retained only 22% and 48% activity, respectively, at 10 mM Fe^3+^. In contrast, Co^2+^, Ni^2+^, Cu^2+^, and Al^3+^ strongly inhibited most enzymes, except for EstEk06, which retained 94–123% activity with Al^3+^, and EstEk07, which maintained 79–104% activity with Co^2+^ and 100–141% with Al^3+^.

Inhibitor studies showed that 10 mM PMSF significantly inhibited EstEk01–07 (12–64% activity), indicating that a serine residue involved in the catalytic triad was modified by PMSF [42, 43]. In contrast, EDTA had no significant effect (81–113% activity), suggesting that EstEk01–07 are not metalloenzymes [44, 45]. All enzymes remained stable in the presence of 1% non-ionic detergents (Triton X-100, Tween 20, and Tween 80). However, 1%SDS significantly decreased activity (53–65%), except for EstEk04 and EstEk05, which retained approximately 80% activity.

The effects of organic solvents were further evaluated by pre-incubating the enzymes in 15% solvent (methanol, ethanol, isopropanol, acetone, acetonitrile, or DMSO) at pH 8.0 and room temperature for 1 h (Table 2). EstEk01–07 displayed high stability in methanol (84–125%), isopropanol (77–107%), and acetonitrile (102–114%), except for EstEk04, which retained 67% activity in acetonitrile. In DMSO, significant inhibition was observed (53–81%). Notably, EstEk01, EstEk04, and EstEk05 retained over 80% residual activity in most solvents except for DMSO or acetonitrile. All enzymes maintained at least 50% activity in all tested solvents, indicating strong organic solvent tolerance.

These results indicate that EstEk01-07 exhibit excellent stability under diverse conditions, including exposure to metal ions, detergents, organic solvents, and thermophilic temperatures. The observed stimulatory effects of Ca^2+^ and Fe^3+^ on enzyme activity suggest possible roles of these ions in stabilizing enzyme conformation or enhancing catalytic efficiency, although the absence of EDTA sensitivity indicates that EstEk01–07 are not classical metalloenzymes [46]. Previous studies have revealed that *Bacillus subtilis* SK320 produces biosurfactants through an esterase-mediated mechanism, with the enzyme playing a critical role in determining bioactivity [47]. In addition, esterases have been shown to metabolize ester-based compounds, such as vitamin A derivatives, thereby regulating both yield and biological activity [48]. Furthermore, esterases are involved in the synthesis of short-chain flavor esters [49]. Overall, these results support the potential of esterases as valuable biocatalysts for various industrial applications, including pharmaceutical and cosmetic production, biopolymer modification, transesterification, and environmental bioremediation [50-52].

## Conclusion

The present findings suggest the promising industrial potential of EstEk01-07, particularly due to their high thermostability, alkaline pH tolerance, and organic solvent resistance. These properties offer advantages over conventional enzymes and synthetic catalysts across various applications. To further elucidate their catalytic mechanisms and optimize their industrial utilization, additional studies, including detailed kinetic analyses, substrate specificity based on full structural analysis, and advanced molecular modeling such as docking studies, are warranted. A deeper understanding of substrate binding, solvent interactions, and optimal reaction conditions will be essential for developing applications such as their use in bioactive substance production or biocatalytic processes. Furthermore, investigations into reaction parameters, enzyme inhibitors, and substrate analogs may provide valuable insights into the mechanisms underlying the unique properties of EstEk01–07 derived from *E. keratini* EPI-7.

## Supplemental Materials

Supplementary data for this paper are available on-line only at http://jmb.or.kr.



## Figures and Tables

**Table 1:** Primers for the GDSL-type lipolytic enzymes from *E. keratini* EPI-7 used for cloning.

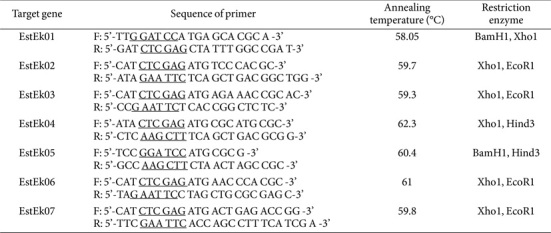

**Table 2:** Effects of organic solvents on recombinant EstEk01-07.

Organic solvents	Residual activity (%)^a,b^ (15%, v/v)
Enzyme	EstEk01	EstEk02	EstEk03	EstEk04	EstEk05	EstEk06	EstEk07
None^c^	100 ± 2.0	100 ± 3.2	100 ± 0.9	100 ± 1.1	100 ± 5.1	100 ± 0.5	100 ± 2.1
Methanol	125 ± 0.8	103 ± 4.1	84 ± 2.3	84 ± 3.0	113 ± 1.7	112 ± 1.2	92 ± 4.3
Ethanol	89 ± 1.5	56 ± 2.2	65 ± 1.0	84 ± 2.2	81 ± 4.1	65 ± 2.4	73 ± 2.9
Isopropanol	107 ± 1.3	82 ± 2.7	77 ± 1.3	85 ± 0.7	106 ± 1.2	84 ± 2.8	91 ± 4.2
Acetone	95 ± 4.0	58 ± 3.8	61 ± 0.4	83 ± 3.6	89 ± 5.7	70 ± 3.9	65 ± 3.2
Acetonitrile	114 ± 3.9	114 ± 2.0	103 ± 4.5	67 ± 1.4	108 ± 2.0	102 ± 1.9	102 ± 0.6
DMSO	77 ± 0.9	53 ± 1.2	74 ± 0.3	81 ± 1.1	64 ± 0.4	58 ± 0.1	73 ± 0.7

^a^Purified enzymes were pre-incubated in a reaction solution containing 50 mM Tris-HCl buffer (pH 8.0), and 15% organic solvent at room temperature for 1 h. The residual activity was measured by initiating the reaction with substrate addition under standard conditions.

^b^Values represent the mean ± SD (*n* = 3) relative to control.

^c^The activity measured without organic solvent was defined as 100%.
