# Supplementary material for: Cloning, Expression, and Characterization of GDSL-Type Lipolytic Enzyme Genes from Epidermidibacterium keratini EPI-7 Isolated from Human Skin
Source: J Microbiol Biotechnol. 2025 Aug 6;35:e2504022. doi: 10.4014/jmb.2504.04022 (PMC12351110; doi:10.4014/jmb.2504.04022)
Supplement: Supplementary file 1 [file jmb-35-e2504022-supple.pdf]

Supplementary Figure 1

#	Template	Alignment Coverage	3D Model	Confidence	% i.d.	Template Information
1	c5tieA_	Alignment	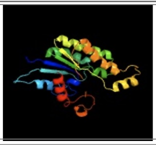	99.9	26	**PDB header:** hydrolase<br>**Chain:** A: **PDB Molecule:** Acyl-CoA thioesterase I<br>**PDBTitle:** x-ray structure of acyl-coa thioesterase i, tesa, mutant m141l/y145k/l146k at ph 7.5 in complex with octanoic acid<br>**PDB Entry:** PDBe RCSB PDBj
2	c8h09B_	Alignment	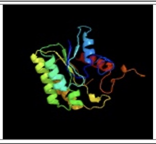	99.7	23	**PDB header:** hydrolase<br>**Chain:** B: **PDB Molecule:** SGNH/GDSL hydrolase family protein<br>**PDBTitle:** structure of the thermolabile hemolysin from vibrio alginolyticus (apo form)<br>**PDB Entry:** PDBe RCSB PDBj
3	c4xvhA_	Alignment	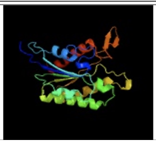	99.6	24	**PDB header:** hydrolase<br>**Chain:** A: **PDB Molecule:** Carbohydrate esterase family 2 (CE2)<br>**PDBTitle:** ;crystal structure of a corynascus thermopiles (myceliophthora fergusii) carbohydrate esterase family 2 (ce2) enzyme plus carbohydrate binding domain (cbd);<br>**PDB Entry:** PDBe RCSB PDBj
4	c4hyqA_	Alignment	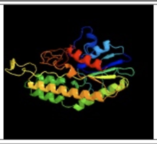	99.9	30	**PDB header:** hydrolase<br>**Chain:** A: **PDB Molecule:** phospholipase A1<br>**PDBTitle:** crystal structure of phospholipase a1 from streptomyces albidoflavus na297<br>**PDB Entry:** PDBe RCSB PDBj
5	c1yzfA_	Alignment	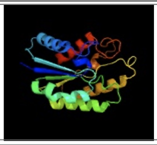	99.8	23	**PDB header:** hydrolase<br>**Chain:** A: **PDB Molecule:** lipase/acylhydrolase<br>**PDBTitle:** crystal structure of the lipase/acylhydrolase from enterococcus faecalis<br>**PDB Entry:** PDBe RCSB PDBj
6	c3kvnA_	Alignment	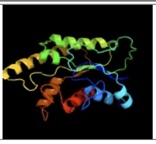	99.4	24	**PDB header:** hydrolase<br>**Chain:** A: **PDB Molecule:** Esterase estA<br>**PDBTitle:** crystal structure of the full-length autotransporter esta from pseudomonas aeruginosa<br>**PDB Entry:** PDBe RCSB PDBj
7	c1yzfA_	Alignment	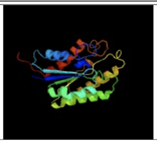	99.9	21	**PDB header:** hydrolase<br>**Chain:** A: **PDB Molecule:** lipase/acylhydrolase<br>**PDBTitle:** crystal structure of the lipase/acylhydrolase from enterococcus faecalis<br>**PDB Entry:** PDBe RCSB PDBj

Fig. S1. Detailed Phyre2 Modeling results of EstEk01–EstEk07.

EstEk01 to EstEk07 are presented in order from 1 to 7. For each enzyme, the Phyre2 modeling results are shown, including the predicted 3D structure, confidence score, alignment coverage, percent identity (% i.d.), and information about the template model used for structure prediction.

Supplementary Figure 2

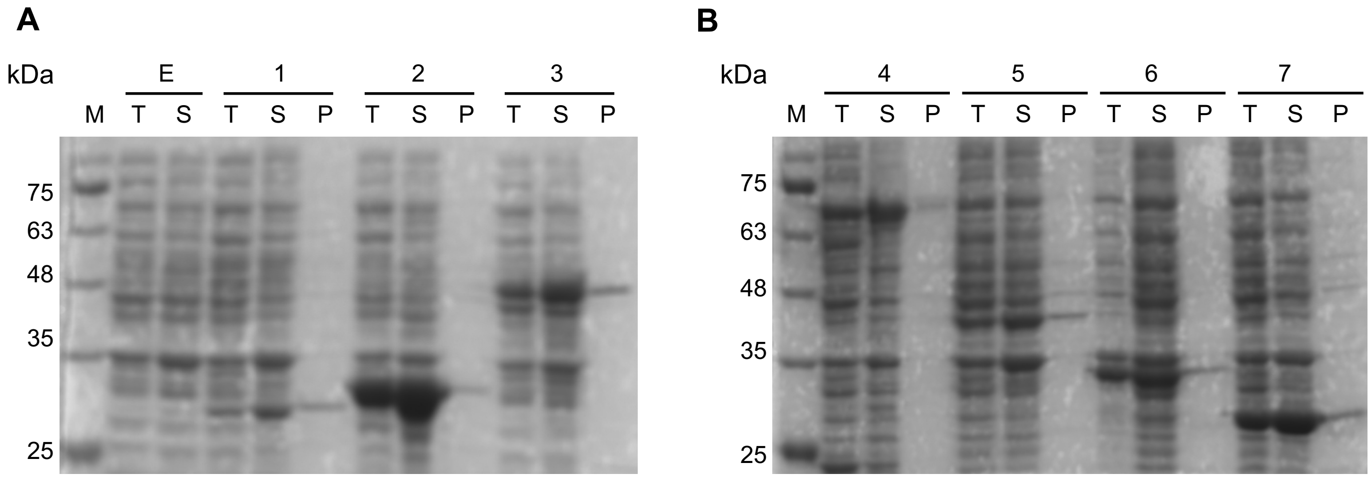

Fig. S2. Expression and Purification of recombinant EstEk01-07 proteins from *E. keratini* EPI-7.

(A) SDS-PAGE analysis of the expression and purification of EstEk01-03. **(B)** SDS-PAGE analysis of the expression and purification of EstEk04-07. lane E, recombinant *E. coli* BL21 harboring the empty vector; lane 1, EstEk01; lane 2, EstEk02; lane 3, EstEk03; lane 4, EstEk04; lane 5, EstEk05; lane 6, EstEk06; lane 7, EstEk07; Lane M, molecular weight marker; Lane T, total cell lysate; Lane S, soluble fraction of cell lysate; Lane P, purified enzyme.

Supplementary Figure 3

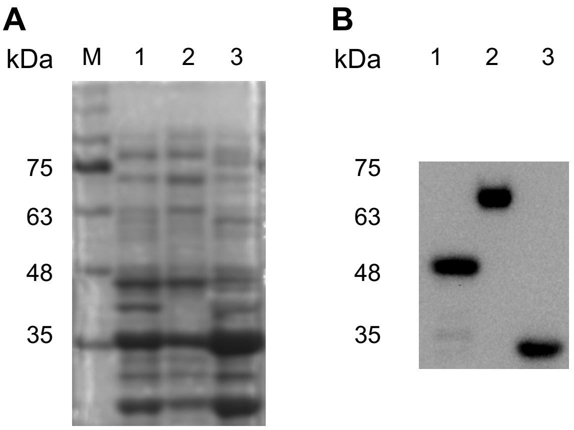

Fig. S3. Periplasmic fraction analysis of recombinant *E. coli* BL21 expressing EstEk03, EstEk04, and EstEk06. **(A)** SDS-PAGE analysis of periplasmic fraction. **(B)** Western blot analysis using an anti-His antibody to detect EstEk03, EstEk04, and EstEk06 in periplasmic fractions. Lane M, molecular size marker; lane 1, periplasmic fraction of recombinant EstEk03; lane 2, periplasmic fraction of recombinant EstEk04; lane 3, periplasmic fraction of recombinant EstEk06.

Supplementary Table 1

Supplementary Table. Signal peptide prediction results for EstEk01-07.

Enzyme	Signal peptide (Sec/SPI)[a]	Lipoprotein signal peptide (Sec/SPII)[b]	TAT signal peptide (Tat/SPI)[c]	TAT lipoprotein signal peptide (Tat/SPII)[d]	Pilin-like signal peptide (Sec/SPIII)[e]	Cleavage site (Probability)
EstEk01	0	0	0	0	0	-
EstEk02	0	0	0	0	0	-
EstEk03	0.8452	0.0033	0.0512	0.0013	0.0004	37-38 (0.761)
EstEk04	0.999	0.0002	0.0002	0.0002	0.0001	25-26 (0.972)
EstEk05	0	0	0	0	0	-
EstEk06	0.0127	0	0.9854	0.0002	0	26-27 (0.665)
EstEk07	0	0	0	0	0	-

[a]Sec/SPI: "standard" secretory signal peptides transported by the Sec translocon and cleaved by Signal Peptidase I (*Lep*)

[b]Sec/SPII: lipoprotein signal peptides transported by the Sec translocon and cleaved by Signal Peptidase II (*Lsp*)

[c]Tat/SPI: Tat signal peptides transported by the Tat translocon and cleaved by Signal Peptidase I (*Lep*)

[d]Tat/SPII: Tat lipoprotein signal peptides transported by the Tat translocon and cleaved by Signal Peptidase II (Lsp)

[e]Sec/SPIII: Pilin and pilin-like signal peptides transported by the Sec translocon and cleaved by Signal Peptidase III (*PilD*/*PibD*)
